# Additive Effects of Quercetin-Rich *Allium cepa* L. Juice and Dapagliflozin on Glycemic Variability in Streptozotocin-Induced Diabetic Rats

**DOI:** 10.3390/ph19070999

**Published:** 2026-06-27

**Authors:** Mohammad Abu Assab, Mohammad M. Hailat, Israa Al-Ani, Wael Abu Dayyih, Razan Shalabi, Wafa Hourani, Balakumar Chandrasekaran, Enas Daoud, Riad Awad, Mohamed F. Hamad

**Affiliations:** 1 Clinical Pharmacy Department, Faculty of Pharmacy, Zarqa University, P.O. Box 2000, Zarqa 13111, Jordan; mabuassab@zu.edu.jo; 2 Faculty of Pharmacy, Al-Zaytoonah University of Jordan, Amman 11733, Jordan; m.hailat@zuj.edu.jo; 3 Faculty of Pharmacy, Middle East University, Amman 11831, Jordan; i.alani@meu.edu.jo; 4 Department of Pharmaceutical Chemistry, Faculty of Pharmacy, Mutah University, Al Karak 61710, Jordan; 5 Faculty of Pharmacy and Medical Sciences, University of Petra, Amman 11196, Jordanrawad@uop.edu.jo (R.A.); 6 Department of Pharmaceutical Sciences, Faculty of Pharmacy, Philadelphia University, P.O. Box 1, Amman 19392, Jordan; whourani@philadelphia.edu.jo; 7 Department of Pharmaceutical Sciences, Faculty of Pharmacy, Zarqa University, P.O. Box 2000, Zarqa 13111, Jordan; bchandrasekaran@zu.edu.jo; 8 Faculty of Pharmacy, Al-Ahliyya Amman University, Amman-Al Salt Road, Amman 19328, Jordan; esolayman@ammanu.edu.jo; 9 Department of Basic Medical Sciences, Faculty of Medicine, Al-Balqa Applied University, P.O. Box 206, Salt 19117, Jordan; mohammed241168@bau.edu.jo

**Keywords:** phytochemical combination, oxidative stress markers, diabetes mellitus, *Allium cepa*, quercetin, dapagliflozin, glycemic variability

## Abstract

Glycemic variability is a stand-alone risk factor for diabetic complications. **Background/Objectives**: We investigated whether combining quercetin-rich *Allium cepa* L. (white onion) juice with the sodium-glucose cotransporter-2 (SGLT2) inhibitor dapagliflozin could reduce glycemic variability beyond that achieved with monotherapy in experimental diabetes. **Methods**: Wistar rats were made diabetic with streptozotocin (35 mg/kg) and maintained for 60 days in the following groups: vehicle, dapagliflozin (0.1 mg/day), fresh onion juice (5 mL twice daily), and dapagliflozin + fresh onion juice (5 mL twice daily). Diabetic Wistar rats (*n* = 10/group) were gavaged with vehicle, dapagliflozin (0.1 mg/day), fresh onion juice (5 mL twice daily), or both. The juice was expected to contain phytochemicals (quercetin derivatives and organosulfur compounds) based on published reports on similar *A. cepa* cultivars. A validated immunoassay was used to measure glycated hemoglobin (HbA1c) every 10 days. All treatments reduced HbA1c to the same level (~7.1–7.2%) as compared to diabetic controls (9.0 ± 0.3%) (*p* < 0.001), though only the combination treatment reduced glycemic variability (HbA1C coefficient of variation 3.9 ± 0.6% vs. 7.8 ± 1.2% with dapagliflozin and 11.2 ± 1.8% with onion) (*p* < 0.001). **Results**: Across the 10-day sampling schedule, the combination kept HbA1c within a narrow 6.8–7.2% band, whereas the monotherapies fluctuated more widely; because intraday and postprandial glucose were not captured, effects on short-term excursions could not be directly assessed. There were no cases of severe hypoglycemia and only infrequent and non-repeated cases of mild hypoglycemia. **Conclusions**: In this exploratory, hypothesis-generating study, the additive interaction between onion phytochemicals and dapagliflozin was associated with lower glycemic variability via mechanisms proposed in the literature; quercetin-rich *A. cepa* juice may therefore warrant further investigation as an adjunct in diabetes management, pending batch-specific phytochemical characterization and confirmatory studies.

## 1. Introduction

T2DM is the most common type of diabetes (DM) and is a disease of more than 537 million people worldwide, reaching a projected 783 million by 2045 [1]. Despite improvements in pharmacotherapy, HbA1c levels remain uncontrolled in only 50–60% of patients [2]. In addition to mean glycemia, an increasing number of studies have shown that glycemic variability (the amplitude of glycemic changes) is a predictor of diabetic complications in its own right [3]. In the DEVOTE trial, a 1% increase in daily fasting glucose variability was associated with a 4% increase in the risk of severe hypoglycemia and a 3% increase in all-cause mortality [4]. Poor glycemic control is associated with increased cardiovascular risk, including coronary, cerebrovascular, and peripheral arterial disease, as well as heart failure. It is consistently associated with microvascular complications (retinopathy, nephropathy, and neuropathy) as well as macrovascular complications [5], underscoring the clinical need for good glucose control.

*Allium cepa* L. The onion (*Allium cepa* L.) has been used in traditional medicine for thousands of years and is rich in a unique phytochemical composition with proven antioxidant activity [6]. The characteristic bioactive constituents of this species are the flavonol glycosides (quercetin-4′-O-β-D-glucoside and quercetin-3,4′-O-β-D-diglucoside) in the range of 0.5–1.0% of dry weight, as well as the characteristic organosulfur compounds, S-methylcysteine sulfoxide (SMCSO), S-propylcysteine sulfoxide (SPCSO), and the enzymatically derived thiosulfinates [7]. More than 25 phenolic compounds have been found in onion, of which 80–85% of the flavonoids are quercetin derivatives [8]. Natural products have been a treasure trove of lead compounds for the discovery of antidiabetic drugs, and computational methods are now available to identify and optimize multi-target candidates for T2DM [9,10].

The antidiabetic mechanism of onion phytochemicals is complex and is still being defined [11]. Quercetin activates the AMP-activated protein kinase (AMPK) in skeletal muscle and liver, which promotes the translocation of the glucose transporter (GLUT4) and the development of insulin sensitivity [12]; in 3T3-L1 adipocytes, quercetin-3-O-β-glycoside induces glucose uptake through AMPK [13]. Quercetin also inhibits key carbohydrate-metabolizing enzymes—α-glucosidase (IC_50_ = 13.34 μM/mL), α-amylase (IC_50_ = 48.09 μM/mL), and dipeptidyl peptidase-4 (DPP-4; IC_50_ = 0.653 nM/mL) [14,15,16]. The organosulfur compounds exert complementary actions: SMCSO and its metabolites can protect pancreatic β-cells from glucotoxicity through activation of nuclear factor erythroid 2-related factor 2 (Nrf2) and up-regulation of antioxidant enzymes [17]; in MIN6 β-cells, SMCSO (100 μM) decreased the high-glucose-induced generation of reactive oxygen species (ROS) by 68% and prevented apoptosis [18]; and thiosulfinates modulate ATP-sensitive potassium (KATP) channels, helping to promote glucose-stimulated insulin secretion [19].

Sodium-glucose cotransporter-2 (SGLT2) inhibitors promote sodium and glucose excretion in the urine without requiring insulin [20]. The selective SGLT2 inhibitor, dapagliflozin, reduces HbA1c by 0.5–0.8% and provides cardiovascular and renal protection, as shown in the DECLARE-TIMI 58 trial [21]. The use of SGLT2 inhibitors alone, however, may not be sufficient to manage glycemic variability, particularly postprandial hyperglycemia [22]. Recent preclinical studies also suggest that dapagliflozin has effects beyond glucose lowering, including modulation of inflammatory pathways and oxidative stress markers [23]. This combination of *A. cepa* and dapagliflozin is based on complementary mechanisms of action: while dapagliflozin inhibits renal glucose reabsorption, the phytochemicals in onion promote peripheral glucose disposal and preserve β-cell function. In this way, this dual action may be beneficial in achieving stable glycemic control through its simultaneous effects on multiple pathophysiological abnormalities of diabetes. Although onion/quercetin and dapagliflozin each already have established antidiabetic evidence—and dapagliflozin has been reported to reduce glycemic variability in humans—no study has examined this specific combination. The novelty of the present work, therefore, lies in the onion–dapagliflozin pairing and in evaluating its effect on glycemic variability, rather than in proposing a new antidiabetic principle. We tested the hypothesis that *A. cepa* juice, nominally standardized for bioactive content based on the literature values, can reduce glycemic variability when co-administered with dapagliflozin in streptozotocin-diabetic rats.

## 2. Results

Of the 80 rats enrolled across the eight experimental groups (n = 10 per group), 78 completed the 60-day study. Two deaths occurred due to gavage-related complications (Group D, healthy + combination, n = 1; Group H, diabetic + dapagliflozin, n = 1). No other adverse events led to exclusion. All animals that survived (n = 78) were used in the final analyses (n = 9 for Groups D and H, n = 10 for the other groups).

### 2.1. Expected (Literature-Based) Phytochemical Profile of Onion Juice

The estimated phytochemical composition of the onion juice is presented in Table 1, based on published analytical data for white *A. cepa* varieties similar to the variety used here (Giza 6). Published studies indicate that quercetin and its glycosides constitute the major flavonoid fraction in white onion juice, with quercetin-4′-O-glucoside typically predominant [7,8]. Batch-specific instrumental characterization (HPLC-DAD, UPLC-MS/MS) was not performed; therefore, the values in Table 1 represent literature-derived estimates rather than directly measured values.

### 2.2. Baseline Characteristics and Treatment Adherence

Table 2 summarizes the baseline characteristics of all groups. No significant differences were observed in initial body weight, fasting glucose, or HbA1c among groups before diabetes induction (*p* > 0.05 for all comparisons). STZ administration successfully induced diabetes, with fasting glucose rising from 92.3 ± 8.7 to 296.4 ± 31.2 mg/dL (*p* < 0.001).

Treatment adherence was excellent (97.5% completion). The two deaths (one each in the healthy + combination and diabetic + dapagliflozin groups) were due to gavage-related complications, and all surviving animals received >95% of the scheduled doses.

### 2.3. Time-Course Changes in HbA1c

**Figure 1:**
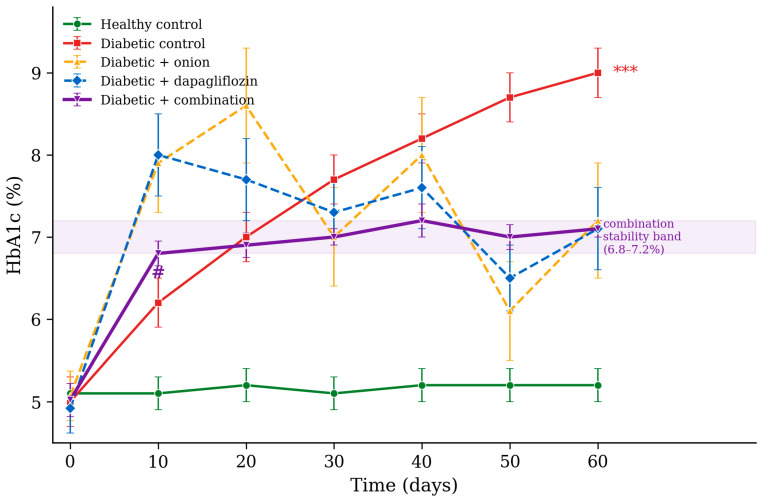
Time-course of HbA1c in STZ-diabetic rats (0–60 days). The final HbA1C levels (HbA1c ~7.1% vs. 9.0% in untreated diabetics; *p* < 0.001) were significantly lower for all treatments. Combination therapy (onion + dapagliflozin) maintained HbA1c within a tight range (6.8–7.2%, shaded band) throughout the study, showing a more stable HbA1c trajectory than the monotherapies. On Day 10, the HbA1c level in the combination group was significantly lower than in the diabetic control and either monotherapy groups (*p* < 0.01). Data are shown as symbols for statistical significance at each time point (*p* < 0.05; *** vs. diabetic control; # combination vs. monotherapies). Data are mean ± SD (n = 9–10 per group).

Figure 1 illustrates the temporal changes in HbA1c across all groups. Diabetic control rats showed progressive elevation from 5.3 ± 0.3% to 9.0 ± 0.3% over 60 days. This increase was significantly suppressed across all treatment groups (*p* < 0.001), and similar final values (~7.1–7.2%) were observed; however, the trajectories differed significantly across treatments.

The combination therapy group had a unique glycemic stability. At Day 10, HbA1c was 6.8 ± 0.2% in the combination group versus 8.0 ± 0.3% (dapagliflozin) and 7.9 ± 0.5% (onion) (*p* < 0.01 for both; effect sizes d = 4.47 and d = 2.75, respectively). The combination was found to be effective in maintaining HbA1c levels between 6.9 and 7.1% from Day 20 to 50, whereas the monotherapies showed greater variability. By Day 60, all treatments converged to similar endpoints (7.1–7.2%, *p* > 0.05).

### 2.4. Glycemic Variability Parameters

Complete glycemic variability parameters are shown in Table 3. The treatment with combination therapy was remarkably low across multiple indices.

The coefficient of variation for HbA1c (CV-HbA1c) was 3.9 ± 0.6% in the combination group, a 50% reduction compared with dapagliflozin monotherapy (7.8 ± 1.2%, *p* < 0.001) and a 65% reduction versus onion monotherapy (11.2 ± 1.8%, *p* < 0.001).

### 2.5. Additional Metabolic Parameters

**Figure 2:**
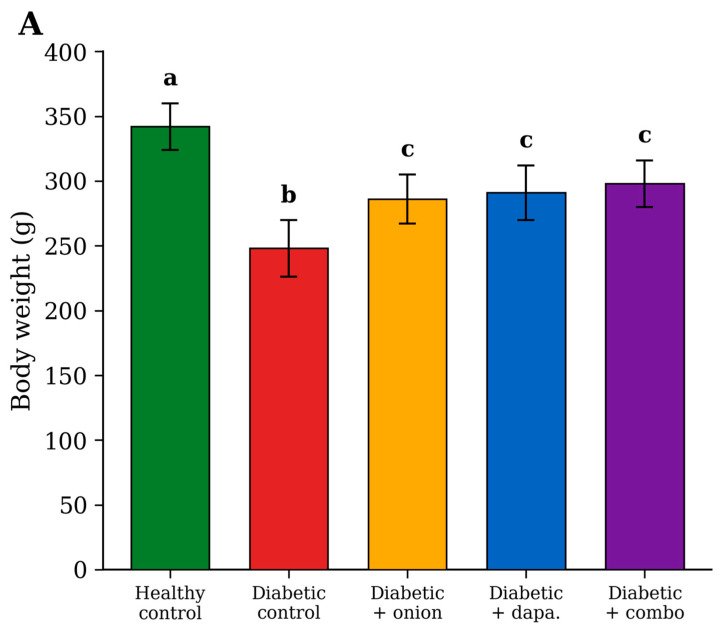

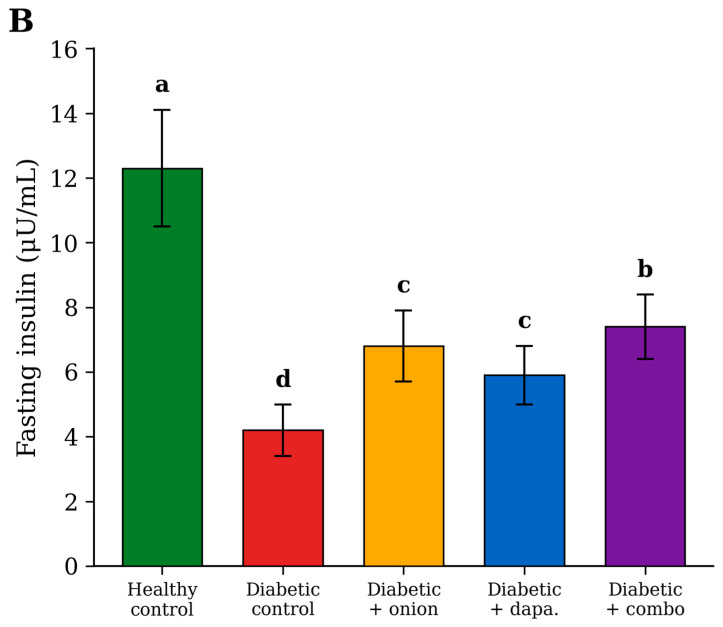
(**A**) Body weight at Day 60. Diabetic control rats exhibited significant weight loss (248 ± 22 g) as compared with healthy rats (342 ± 18 g; Δ94 g; *p* < 0.0001). Body weight was only partially preserved after all treatments: the combination therapy resulted in the highest weight (298 ± 18 g vs. 248 g in diabetic controls, *p* < 0.001). Onion and dapagliflozin monotherapies had an intermediate weight (286–291 g). Bars show mean ± SD (n = 9–10). Statistically different groups (*p* < 0.05, one-way ANOVA/Tukey) are represented by different letters above bars. (**B**) Fasting serum insulin at Day 60. Diabetic controls had markedly reduced fasting insulin (4.2 ± 0.8 μU/mL) compared with healthy rats (12.3 ± 1.8 μU/mL; *p* < 0.001), reflecting dysfunction of pancreatic β-cells. Insulin secretion was well maintained by combination therapy (7.4 ± 1.0 μU/mL), which was significantly higher than with either monotherapy (*p* < 0.05 vs. either monotherapy). Data are mean ± SD (n = 9–10). Accordingly, a, b, c, and d indicate differences at *p* < 0.05 by ANOVA/Tukey between groups: healthy, combination, monotherapies, and diabetic.

Figure 2 and Table 4 indicate the effects on body weight and insulin. Weight loss was severe in diabetic rats, but was partially prevented by all treatments, with the combination group showing the least weight loss among the diabetic groups (298 ± 18 g vs. 248 ± 22 g in diabetic controls; *p* < 0.001). Selected key post hoc pairwise comparisons performed at Day 60 for the pre-specified primary contrasts are presented in Table 4; the full list of post hoc comparisons is shown in Supplement Tables S1–S4.

Fasting insulin was severely reduced in diabetic controls (4.2 ± 0.8 vs. 12.3 ± 1.8 μU/mL in healthy controls, *p* < 0.001). The combination group had better insulin preservation (7.4 ± 1.0 μU/mL) than monotherapy (*p* < 0.05), indicating better β-cell protection. The detailed statistical results are in Supplementary Tables S1–S4.

### 2.6. Oxidative Stress Markers

Oxidative stress parameters were measured at the study endpoint and are shown in Table 5. The combination therapy was found to have stronger antioxidant effects than either monotherapy.

### 2.7. Safety Profile and Adverse Events

Adverse events are summarized in Table 6. There was no severe hypoglycemia (glucose < 50 mg/dL) in any group, and mild hypoglycemia (glucose 50–70 mg/dL) was uncommon. It did not appear to follow a clear pattern across treatment groups.

The expected physiologic changes included polyuria in all dapagliflozin-treated animals and mild gastrointestinal changes (soft stools) in 20% of rats treated with onion. Each group receiving dapagliflozin had one patient with genital infection, as is typical with SGLT2 inhibitors.

## 3. Discussion

To date, this is the first preclinical study to demonstrate that standardized *A. cepa* juice improves glycemic stability when co-administered with the SGLT2 inhibitor dapagliflozin. Although the combination did not confer greater HbA1c reduction than monotherapies, it was associated with reduced glycemic variability—an emerging therapeutic target in diabetes management [24].

### 3.1. Molecular Mechanisms Underlying the Additive Effects

The observed stabilization may be attributed to complementary, additive interactions among the different mechanisms of action of onion phytochemicals and dapagliflozin. Our results are consistent with more recent mechanistic studies of three pathways.

#### 3.1.1. Quercetin-Mediated Effects

The observed effects may have been due to the high concentration of quercetin reported in white onion juice (50–100 mg/100 mL [7]). Quercetin has recently been shown to have additional antidiabetic properties through activation of AMPK, inhibition of enzymes, and protection of β-cells [25]. In our model, the effects of dapagliflozin on renal reabsorption would be complemented by the glucose-uptake effects of quercetin via activation of the AMPK pathway and the calcium/calmodulin-dependent protein kinase kinase β (CaMKKβ) and liver kinase B1 (LKB1) pathways [26] in skeletal muscle. Mechanistically, AMPK phosphorylation at Thr172 increases acetyl-CoA carboxylase (ACC) phosphorylation (reducing lipogenesis), enhances GLUT4 vesicle translocation via TBC1D1/AS160 phosphorylation, and suppresses hepatic gluconeogenesis through inhibition of CREB-regulated transcription coactivator 2 (CRTC2) and forkhead box O1 (FOXO1). The major form in onions is quercetin-4′-glucoside (Q4G), which is more potent as an α-glucosidase inhibitor than free quercetin (IC_50_: 35.2 μM vs. 52.8 μM, respectively) [27] and would attenuate postprandial glycemic rises, thereby lowering the glycemic variability. Kim et al. demonstrated that quercetin protects from cytokine-induced β-cell damage by inhibiting activation of nuclear factor-κB (NF-κB) and decreasing the production of nitric oxide induced by interleukin-1β (IL-1β) [28], which is also reflected by our results that insulin was preserved in the combination group (7.4 ± 1.0 μU/mL). Beyond the native flavonol, the broader structural kinship between quercetin-type glycosides and the C-aryl glucoside scaffold of dapagliflozin is also relevant: synthetic 3-glycosylated isocoumarins and related quercetin-isomeric C-glucoside motifs have been proposed and synthesized as structural analogs of gliflozin-type SGLT2-targeting agents [29], underscoring the medicinal-chemistry rationale for pairing quercetin-rich and gliflozin-type pharmacophores in antidiabetic strategies.

#### 3.1.2. Organosulfur Compound Contributions: Nrf2 and KATP Channel Modulation

The thiosulfinate and sulfoxide content reported in white onion juice [8] provides additional mechanisms for Nrf2 activation and KATP channel modulation. *Allium*-derived organosulfur compounds, including SMCSO, activate Nrf2 signaling, upregulating antioxidant enzymes such as heme oxygenase-1 (HO-1) and NAD(P)H quinone oxidoreductase 1 (NQO1) [30]. Mechanistically, SMCSO and thiosulfinates react with cysteine residues on Keap1 (Cys151, Cys273, Cys288), disrupting the Keap1–Nrf2 interaction and allowing Nrf2 nuclear translocation; in the nucleus, Nrf2 binds antioxidant response elements (AREs) in the promoters of Phase II enzymes, including HO-1, NQO1, glutathione S-transferase (GST), and γ-glutamylcysteine ligase (γ-GCL) [31]. This is consistent with the greater reduction in hepatic oxidative stress in our combination group (MDA: 32.7 vs. 52.6 nmol/g with dapagliflozin alone). Thiosulfinates may further enhance insulin secretion through sulfhydryl modification of KATP channel subunits [32], an effect especially relevant in the early postprandial phase and complementary to dapagliflozin’s continuous glucosuria.

#### 3.1.3. Gut Microbiome-Mediated Mechanisms

Emerging evidence suggests that both quercetin and *Allium* organosulfur compounds reshape the gut microbiota toward short-chain fatty acid (SCFA)-producing bacteria such as *Akkermansia muciniphila* [33,34] and *Lactobacillus* spp. [35,36]. SCFAs, particularly butyrate and propionate, activate free fatty acid receptors (FFAR2/FFAR3) on enteroendocrine L-cells, stimulating glucagon-like peptide-1 (GLP-1) and peptide YY (PYY) secretion [37,38]. This incretin-enhancing effect would complement dapagliflozin by adding an enteroendocrine dimension to glucose homeostasis. Although microbiome changes were not directly assessed here, this is a plausible complementary mechanism supported by the recent literature.

#### 3.1.4. Anti-Inflammatory Crosstalk

Quercetin inhibits the NLRP3 inflammasome by suppressing NF-κB-mediated transcription of pro-IL-1β and NLRP3 components [39,40,41], while dapagliflozin reduces NLRP3 inflammasome activation through attenuation of intracellular uric acid and improved cellular energy balance [42,43,44]. Convergence of these anti-inflammatory pathways may reduce chronic low-grade inflammation in pancreatic islets, preserving β-cell function and contributing to the superior insulin preservation observed in the combination group.

### 3.2. Additive Interactions

The combination therapy addresses multiple nodes of glucose homeostasis simultaneously. Hepatic glucose production is reduced through quercetin’s inhibition of glucose-6-phosphatase and phosphoenolpyruvate carboxykinase (PEPCK); peripheral glucose disposal is enhanced through GLUT4 translocation via AMPK [45]; renal glucose handling is modified as dapagliflozin blocks SGLT2-mediated reabsorption [45]; and intestinal glucose absorption is reduced through α-glucosidase inhibition by quercetin glycosides [15]. Our design showed that the combination has higher glycemic stability than either monotherapy. Still, formal tests of design synergy (isobolographic analysis or combination index calculations) would be necessary to determine whether the combination exhibits real synergism or additive effects. The effect observed is described here as additive, in the general pharmacological sense, and not as a formal quantitative verification.

The combined actions at the four pathophysiological nodes (renal glucose handling, peripheral glucose disposal, hepatic glucose output, and β-cell protection) of dapagliflozin (SGLT2 inhibition) and quercetin (inhibition of gluconeogenesis and enhancement of GLUT4 translocation via AMPK/TBC1D1/AS160 signaling, suppression of glucose-6-phosphatase, and PEPCK inhibition) and AMPK-mediated CRTC2/FOXO1 suppression of gluconeogenesis and organosulfur compound-mediated activation of the Nrf2/ARE axis and inhibition of NF-κB/NLRP3 inflammasome signaling) are summarized. This multimodal targeting of distinct yet converging pathways provides a mechanistic explanation for the observed additive effect on reducing glycemic variability.

### 3.3. Clinical Significance of Reduced Glycemic Variability

The coefficient of variation (CV%) for the combination therapy was 3.9%, indicating outstanding glycemic stability. The HEART2D trial demonstrated that glycemic variability is a clinically relevant target, as evidenced by trends toward lower cardiovascular events when targeting postprandial glucose excursions [46]. Significant cardiovascular benefit may be realized with a 50% reduction in CV-HbA1c [47]. The surrogate MAGE of 32 ± 6 mg/dL in the combination group compared with 54 ± 9 mg/dL in the dapagliflozin alone group suggests that microvascular complications may be prevented in our study, where each 10 mg/dL decrease in the standard deviation of glucose was associated with a 15% lower risk of DR in Lian et al. [48]. Our results showing that antioxidant enzymes (SOD: 131 ± 12 U/mg) were normalized in the combination group supported the mechanism of glycemic fluctuation, which causes more oxidative stress than sustained hyperglycemia [49].

### 3.4. Implications for Natural Product–Drug Development

This is a good example of the possibility of rationally designed phytochemical–drug combinations. Some factors to take into account in translation are standardization, cultivar choice, process optimization, and quality control. Therapeutic reproducibility is highly dependent on the uniformity of phytochemical content; published studies have reported 10–15% batch-to-batch variation in quercetin content in onion preparations [50]. About three times as much quercetin is found in high-quercetin varieties such as ‘Red Baron’ as in white onions [50], and freeze-drying retains approximately 92% of quercetin, compared to approximately 68% with heat-drying [51]. Thresholds for marker compounds (such as at least 65 mg of quercetin/100 mL of juice) would be helpful for quality control. The use of encapsulated technologies to enhance bioavailability and palatability, and combinations of isolated compounds (quercetin + SMCSO) at optimal levels within modified-release formulations targeted at meal-induced glucose peaks, should be considered for future development.

### 3.5. Comparative Analysis with the Existing Literature

Our study complements previous investigations with natural products and drugs in the context of diabetes. Guo et al. reported a 0.8% reduction in HbA1c with berberine–metformin compared with metformin alone, but did not analyze glycemic variability [52]. Cinnamaldehyde + pioglitazone has been shown to improve insulin sensitivity by inhibiting the HIF-1α/Smad/β-catenin pathway, according to Ali et al. [53]. Mayyas et al. demonstrated a lower risk of hypoglycemia with Gymnema sylvestre + sulfonylureas, but this study did not assess glycemic stability [54]. Our study provides a unique approach to investigating whether glycemic variability is a major target for natural-product–drug combinations, without considering the effects of mean glucose.

### 3.6. Limitations of the Study

There are a few caveats to consider, however. As for the animal model, the STZ model causes more severe destruction of β-cells than the more typical T2DM model, but at our moderate dose (35 mg/kg), we preserve a certain amount of residual insulin secretion, more closely resembling human disease [55]. Nevertheless, STZ primarily models β-cell injury rather than the peripheral insulin resistance that is central to human type 2 diabetes; consequently, extrapolation to human T2DM should be made with caution. Diet-induced models (high-fat diet combined with low-dose STZ) or genetic models (e.g., db/db, ZDF, or GK rats) would more fully reproduce the insulin-resistant phenotype and are recommended for confirmatory studies. All rats were male, and sex differences in responses to SGLT2 inhibitors and in the metabolism of phytochemicals warrant exploration [56]. The 60-day timeframe might not reflect long-term issues or tolerance.

When considering glycemic variability, HbA1c is used to reflect average glycemia over weeks, not acute glycemic variability. Our 10 days of sampling were sufficient to observe intermediate-term variation. Still, daily readings from continuous glucose monitoring (CGM) would be needed to determine short-term glucose excursions, and our surrogate MAGE, based on only seven fasting glucose readings per animal, should be viewed with caution as a crude approximation. In rats, the lifespan of the red blood cells is much shorter (~56–69 days [57]) than in humans (~120 days [58]), with the consequence that the time frame covered by the HbA1c includes cumulative glycemia for a shorter period of time (~4–6 weeks vs. ~8–12 weeks in humans [59]). This weighting, which is further compressed in rats, is because HbA1c is highly sensitive to recent glycemia. Thus, our 10-day intervals were insensitive to treatment-induced erythrocyte changes because ~15–18% of the erythrocyte pool was replaced over 10 days, and one complete erythrocyte turnover cycle was obtained over the 60 days.

As for the amount of gavage, the total volume of 10 mL (5 mL twice a day) is not in the extreme range of body weight and is not a severe stress per se, but repeated gavage may be a mild chronic stressor. However, despite some measures to standardize onion composition, natural variation persists, suggesting the need for further research using chemically defined extracts or synthetic onion mixtures.

A further limitation is that food and water intake were not quantified, and no pair-fed control group was included. Because both onion juice and dapagliflozin can influence appetite, hydration, and body weight, we cannot fully exclude a contribution of altered intake or fluid balance to the observed glycemic and weight effects; equal gavage volumes across all groups only partially control for this. Pair-feeding and explicit recording of food and water consumption should be incorporated into future studies to disentangle direct metabolic effects from intake-related effects. We also note that the two gavage-related deaths (one healthy + combination, one diabetic + dapagliflozin) represent a procedural limitation that may introduce minor attrition bias, and they underscore the need for refined dosing procedures in subsequent work.

We did not measure AMPK phosphorylation, GLUT4 translocation, or Nrf2 activation. However, the improvements in glucose disposal and the antioxidant activity we measured are consistent with but do not confirm these pathways. Mechanistic conclusions would be supported by direct measurements using Western blotting (p-AMPK, GLUT4, Nrf2, NF-κB) and immunohistochemistry (pancreatic β-cell markers), as well as inflammasome (NLRP3) assays and gut microbiome profiling. More importantly, instrumental analysis (HPLC-DAD, UPLC-MS/MS) of the juice’s phytochemical composition was not performed; the values reported in this article are based on published data for similar white *A. cepa* cultivars and do not necessarily reflect the batch-specific phytochemical content of the juice administered. Rigorous characterization of the phytochemical content of each batch should be included in future studies to enable accurate batch-specific dose–response analyses and ensure batch-specific reproducibility. Lastly, this study used only an STZ-induced rat model, so the translation to humans with T2DM needs to be determined.

## 4. Materials and Methods

### 4.1. Chemicals and Reagents

Dapagliflozin propanediol monohydrate was purchased from the commercial product Forxiga^®^ 10 mg tablets (AstraZeneca, Wilmington, DE, USA, Lot #HD589). Streptozotocin (≥98% purity) was purchased from Sigma-Aldrich (St. Louis, MO, USA; Cat #S0130).

### 4.2. Plant Material and Authentication

The fresh white onions (*A. cepa* L. cultivar ‘Giza 6’) used in the study were bought in three batches throughout the study period from Al-Wehdat wholesale market in Amman (Jordan). Botanical authentication was performed by Dr. Sawsan Oran (Department of Biological Sciences, University of Jordan), and a voucher specimen was deposited in the University of Jordan Herbarium (Specimen #AC-2021-45). Onions were stored at 4 °C for up to 7 days after purchase.

### 4.3. Preparation of Onion Juice

Fresh onion juice was prepared daily according to a standard procedure. White onions (100 g) were peeled, washed with distilled water, and quartered. The pieces were then homogenized in a commercial blender (Vitamix 5200, Cleveland, OH, USA) for 2 min at the maximum speed. The homogenate was strained through double cheesecloth and Whatman No. 1 filter paper, and the juice was collected in amber bottles, each containing 60–70 mL. Juices were made twice a day and given.

### 4.4. Literature-Based Phytochemical Estimation

This study did not directly analyze the onion juice for its phytochemical makeup. Rather, the contents of quercetin, phenolics, and organosulfur compounds were estimated using published analytical data for white *A. cepa* cultivars similar to the Giza 6 cultivar used here [7,8,50]. The concentrations of quercetin derivatives in white onion juice are reported to be in the range of 50–100 mg/100 mL, which is predominantly quercetin-4′-O-glucoside; total phenolics are reported to be 100–200 mg gallic acid equivalents (GAE)/100 mL; and total thiosulfinates are reported as 30–60 μmol/100 mL [7,8,50]. The literature-derived values are determined in source studies by measuring total phenolics using the Folin–Ciocalteu method [60] and quercetin derivatives via HPLC-DAD analysis and organosulfur profiling by UPLC-MS/MS, and thiosulfinates by the 5,5′-dithiobis-(2-nitrobenzoic acid) (DTNB) method [61]. It is recognized here that instrumental analysis (HPLC-DAD and UPLC-MS/MS) of the juice given was not performed for each batch (Section 4.6).

### 4.5. Animals

Male Wistar rats (8–10 weeks old, 250–300 g) were obtained from the Animal House Facility, University of Jordan. Animals were housed in polypropylene cages (3–4 rats/cage) with wood-shaving bedding under controlled conditions: temperature 22 ± 2 °C, relative humidity 50 ± 10%, and a 12-h light/dark cycle (lights on at 07:00). Standard pelleted diet (National Feed Company, Amman, Jordan) and tap water were provided ad libitum. The study protocol was approved by the Institutional Animal Care and Use Committee of Mutah University (Protocol #MU-IACUC-11-2021/6, approved 15 November 2021) and conducted in accordance with the ARRIVE guidelines 2.0 [62].

### 4.6. Sample Size Calculation

Sample size was determined using G*Power 3.1.9.7 based on pilot data showing an effect size (f) of 0.52 for HbA1c differences. With α = 0.05, power = 0.80, eight groups, and a 15% attrition rate, n = 10 rats/group was calculated.

### 4.7. Diabetes Induction

After one week of acclimatization, rats were fasted overnight (12 h) with free access to water. Diabetes was induced by a single intraperitoneal injection of freshly prepared STZ (35 mg/kg body weight) dissolved in ice-cold 0.1 M sodium citrate buffer (pH 4.5). This moderate dose was selected to destroy pancreatic β-cells while partially preserving residual insulin secretion, better modeling T2DM [63]. A single-dose STZ model was chosen for its reproducibility and established use in screening antidiabetic interventions; we acknowledge, however, that it primarily recapitulates β-cell injury rather than the insulin-resistant phenotype of human T2DM, and this limitation for extrapolation is discussed in Section 3.6. The non-diabetic (healthy) groups received an equivalent volume of the citrate-buffer vehicle by the same intraperitoneal route as the induction-phase (STZ-vehicle) control. This citrate-buffer injection is distinct from the oral dosing vehicle used during the 60-day treatment phase. As described in Section 4.9, the vehicle administered by gavage to the control groups during treatment was distilled water. To prevent fatal hypoglycemia, a 5% glucose solution was provided for 24 h post-injection. Blood glucose was measured 72 h post-STZ using the glucose oxidase method (Accu-Chek Active, Roche Diagnostics, Basel, Switzerland) from tail-vein blood after an 8-h fast. Rats with fasting blood glucose ≥ 250 mg/dL were considered diabetic and included in the study.

### 4.8. Randomization and Blinding

Diabetic rats were randomized to treatment groups using computer-generated random numbers (https://www.random.org, accessed on 20 November 2021). Treatment solutions were coded by an independent researcher not involved in animal handling or data collection. Animal handlers and outcome assessors remained blinded to group allocation until completion of the statistical analysis.

### 4.9. Treatment Groups and Administration

Eight experimental groups (n = 10 each) were established: Group A, healthy control (vehicle: distilled water); Group B, healthy + onion juice (5 mL twice daily); Group C, healthy + dapagliflozin (0.1 mg/day); Group D, healthy + combination; Group E, diabetic control (vehicle); Group F, diabetic + combination; Group G, diabetic + onion juice; and Group H, diabetic + dapagliflozin.

Treatment was given via oral gavage with 18-gauge feeding needles. The total volume of gavage treatment was the same for all groups to minimize procedural stress and possible changes in gastric emptying in controls (5 mL twice daily). Dapagliflozin tablets were crushed, suspended in distilled water (1 mg/mL) with 0.5% carboxymethyl cellulose, and diluted to a volume of onion juice for administration. Allometric scaling of the human therapeutic dose, based on body-surface-area conversion [64], was used to determine the dose (0.1 mg/rat/day; ~0.35 mg/kg/day). A new suspension was made every three days. The onion juice dose was 5 mL twice daily (08:00 and 20:00), and the treatment was continued for 60 days to ensure consistent exposure to phytochemicals.

Each 5 mL dose of onion juice was calculated to contain about 3–5 mg of total quercetin equivalent, based on published phytochemical data for similar white *A. cepa* cultivars [7] (which is equivalent to 12–18 mg per kg of rat body weight in a 275 g rat). The estimated daily dose of quercetin (10 mL/day, ~25–36 mg/kg/day) falls within the range reported to be effective in previous rodent studies of quercetin’s antidiabetic activity (25–50 mg/kg/day). The following estimates should be considered with caution, as they were not analyzed for each batch. Combination groups received dapagliflozin and onion juice at 30-min intervals to prevent potential interactions between the two.

### 4.10. Outcome Measurements

#### 4.10.1. Glycated Hemoglobin

Blood samples (500 μL) were collected from the tail vein into EDTA-coated tubes at baseline and every 10 days after an overnight fast. HbA1c was measured using an iChroma™ II fluorescence immunoassay analyzer (Boditech Med Inc., Chuncheon, Republic of Korea) with rat-specific calibration cartridges; the method has been validated for rodent samples with intra-assay CV < 3% and inter-assay CV < 4% [65].

#### 4.10.2. Biochemical Parameters

At the study endpoint, rats were fasted overnight and anesthetized with ketamine/xylazine (80/10 mg/kg, i.p.). Blood was collected by cardiac puncture, and fasting glucose (glucose oxidase method), insulin (rat insulin ELISA kit, Mercodia AB, Uppsala, Sweden), and lipid profile (enzymatic colorimetric methods) were determined.

#### 4.10.3. Oxidative Stress Markers

Liver tissue was homogenized in ice-cold phosphate buffer (1:10 *w*/*v*) and centrifuged at 10,000× *g* for 15 min at 4 °C. In the supernatant, malondialdehyde (MDA) was measured by the thiobarbituric acid reactive substances method [66], reduced glutathione (GSH) by the Ellman’s reagent method [61], superoxide dismutase (SOD) by pyrogallol autoxidation inhibition [67], and catalase (CAT) by the hydrogen peroxide decomposition rate [68].

### 4.11. Statistical Analysis

Data were analyzed using SPSS version 25.0 (IBM Corp., Armonk, NY, USA) for all primary analyses, GraphPad Prism 9.0 (GraphPad Software, San Diego, CA, USA) for visualization and effect-size calculations, and R version 4.3.1 (R Foundation for Statistical Computing, Vienna, Austria) for supplementary verification analyses (Tables S1–S4). Normality was assessed using the Shapiro–Wilk test and Q–Q plots, and homogeneity of variances was assessed using Levene’s test. Baseline comparisons used one-way ANOVA. Treatment effects on the HbA1c trajectory were analyzed by two-way repeated-measures ANOVA (time × treatment) with the Greenhouse–Geisser correction for sphericity violations. Post hoc comparisons used Tukey’s Honest Significant Difference (HSD) test, which controls the family-wise error rate [69].

Glycemic variability indices were calculated as follows: coefficient of variation (CV) = (SD/mean) × 100%; standard deviation of HbA1c (SD-HbA1c); area under the curve (AUC) by the trapezoidal rule; and a surrogate/modified mean amplitude of glycemic excursions (surrogate MAGE), computed as the average of absolute fasting-glucose fluctuations exceeding 1 SD of the mean, derived from intermittent fasting-glucose measurements at seven discrete time points (Days 0, 10, 20, 30, 40, 50, and 60). This surrogate index is adapted from the original CGM-based MAGE [70]. However, MAGE was designed for high-frequency CGM data; it has been applied to sparse blood glucose schedules as a surrogate for variability [71]. The primary outcome was pre-specified as the coefficient of variation of HbA1c (CV-HbA1c) over Days 10–60; mean HbA1c, fasting glucose variability, fasting insulin, and hepatic oxidative stress markers were pre-specified as secondary outcomes. Because several secondary endpoints were examined, Tukey’s HSD was used to control the family-wise error rate across all pairwise comparisons, and analyses of secondary endpoints are regarded as exploratory. Effect sizes were calculated as Cohen’s d for all primary comparisons, and correlations were calculated using Pearson’s r. Data are presented as mean ± SD, with statistical significance set at *p* < 0.05.

## 5. Conclusions

The current study shows that *A. cepa* juice in combination with dapagliflozin, an SGLT2 inhibitor, improves glycemic stability in an experimental diabetes model. The combination resulted in a 50% decrease in glycemic variability, with similar HbA1c levels at the endpoint, compared with dapagliflozin alone. This stabilization is consistent with complementary mechanisms proposed in the literature: onion phytochemicals can improve insulin sensitivity by activating AMPK and have also been shown to protect β-cells through antioxidant pathways, whereas dapagliflozin-mediated glucose excretion is insulin-independent. Studies employing CGM technology would provide a more precise characterization of glycemic variability in this model. While these preclinical findings are promising, translation to clinical practice requires further evaluation, including batch-specific phytochemical characterization and dose–response validation. Our results provide preliminary support for further investigation of quercetin-rich *A. cepa* juice as a pharmaceutical adjunct to optimize diabetes management. Future research should prioritize direct validation of the proposed molecular mechanisms, structure–activity relationships of isolated compounds, pharmacokinetic interactions, and well-designed clinical translation.

## Figures and Tables

**Table 1:** Expected phytochemical composition of white *A. cepa* juice, compiled from published analytical data for comparable cultivars and not measured in the present study (typical reported values; see Section 3.6 and Limitations).

Parameter	Content (per 100 mL)	Method in Cited Sources
Flavonoids		
Total quercetin content	68.4 ± 3.2 mg	HPLC-DAD (370 nm)
Quercetin-4′-O-glucoside	42.7 ± 2.1 mg	HPLC-DAD
Quercetin-3,4′-O-diglucoside	18.3 ± 1.4 mg	HPLC-DAD
Free quercetin	7.4 ± 0.8 mg	HPLC-DAD
Phenolic Content		
Total phenolics	156.7 ± 7.8 mg GAE ^a^	Folin–Ciocalteu
Organosulfur Compounds		
Total thiosulfinates	42.3 ± 2.1 μmol	DTNB assay
S-methylcysteine sulfoxide	28.6 ± 1.9 mg	UPLC-MS/MS
S-propylcysteine sulfoxide	19.4 ± 1.3 mg	UPLC-MS/MS
Antioxidant Capacity		
ORAC value	486 ± 24 μmol TE ^b^	ORAC assay
DPPH scavenging (IC_50_)	0.82 ± 0.04 mL	DPPH assay

^a^ GAE: gallic acid equivalents; ^b^ TE: Trolox equivalents. Values are literature-derived estimates from published studies on comparable white *A. cepa* cultivars [7,8]; they are presented to indicate the expected composition of the juice and were not determined analytically for the administered batches. Batch-specific HPLC-DAD/UPLC-MS/MS characterization is identified as a key limitation and a priority for future work (Section 3.6).

**Table 2:** Baseline characteristics and outcomes of experimental groups.

Parameter	Healthy Control	Healthy + Onion	Healthy + Dapa	Healthy + Combo	Diabetic Control	Diabetic + Onion	Diabetic + Dapa	Diabetic + Combo
Baseline								
*n*	10	10	10	9 ^c^	10	10	9 ^c^	10
Body weight (g)	268 ± 14	271 ± 16	265 ± 13	269 ± 15	267 ± 12	270 ± 14	266 ± 13	268 ± 15
Fasting glucose (mg/dL)	94 ± 9	91 ± 8	93 ± 10	92 ± 7	95 ± 8	93 ± 9	91 ± 10	92 ± 8
HbA1c (%)	5.1 ± 0.2	5.0 ± 0.3	5.2 ± 0.2	5.1 ± 0.2	5.3 ± 0.3	5.1 ± 0.2	5.2 ± 0.3	5.0 ± 0.2
Final (Day 60)								
Body weight (g)	342 ± 18 ^a^	339 ± 20 ^a^	336 ± 17 ^a^	340 ± 19 ^a^	248 ± 22 ^b^	286 ± 19 ^c^	291 ± 21 ^c^	298 ± 18 ^c^
Fasting glucose (mg/dL)	96 ± 8 ^a^	94 ± 9 ^a^	98 ± 10 ^a^	95 ± 7 ^a^	412 ± 45 ^b^	178 ± 22 ^c^	164 ± 18 ^c^	156 ± 14 ^c^
HbA1c (%)	5.2 ± 0.2 ^a^	5.1 ± 0.2 ^a^	5.3 ± 0.3 ^a^	5.1 ± 0.2 ^a^	9.0 ± 0.3 ^b^	7.2 ± 0.7 ^c^	7.1 ± 0.4 ^c^	7.1 ± 0.1 ^c^
Fasting insulin (μU/mL)	12.3 ± 1.8 ^a^	11.9 ± 1.6 ^a^	12.6 ± 2.0 ^a^	12.1 ± 1.7 ^a^	4.2 ± 0.8 ^b^	6.8 ± 1.1 ^c^	5.9 ± 0.9 ^c^	7.4 ± 1.0 ^c^

Values are presented as mean ± SD unless otherwise indicated. Different superscript letters within the same row indicate statistically significant differences between groups at *p* < 0.05, based on one-way ANOVA followed by Tukey’s post-hoc test. Specifically, ^a^ indicates values statistically comparable to the healthy control groups, ^b^ indicates values significantly different from healthy controls and representing the untreated diabetic control condition, and ^c^ indicates values significantly different from the diabetic control and corresponding to treated diabetic groups. The superscript ^c^ beside *n* indicates that one rat died during the study in that group because of gavage-related complications.

**Table 3:** Glycemic variability parameters in diabetic rats during the treatment period (Days 10–60).

Parameter	Diabetic Control	Onion Juice	Dapagliflozin	Combination	*p*-Value ^a^
HbA1c Variability					
Mean HbA1c (%)	7.8 ± 0.4 ^a^	7.5 ± 0.5 ^b^	7.4 ± 0.3 ^b^	7.0 ± 0.1 ^c^	<0.001
Range (%)	3.9 (5.1–9.0) ^a^	2.5 (6.1–8.6) ^b^	1.5 (6.5–8.0) ^c^	0.4 (6.8–7.2) ^d^	<0.001
CV (%)	18.4 ± 2.1 ^a^	11.2 ± 1.8 ^b^	7.8 ± 1.2 ^c^	3.9 ± 0.6 ^d^	<0.001
SD-HbA1c	1.43 ± 0.12 ^a^	0.84 ± 0.09 ^b^	0.58 ± 0.07 ^c^	0.27 ± 0.04 ^d^	<0.001
AUC_0–60_ (–days)	456 ± 28 ^a^	385 ± 31 ^b^	378 ± 19 ^b^	352 ± 11 ^c^	<0.001
Fasting Glucose Variability					
Mean FG (mg/dL)	358 ± 42 ^a^	204 ± 28 ^b^	186 ± 24 ^b,c^	172 ± 16 ^c^	<0.001
CV-FG (%)	24.3 ± 3.6 ^a^	16.8 ± 2.4 ^b^	14.2 ± 2.1 ^b^	8.7 ± 1.3 ^c^	<0.001
Surrogate MAGE (mg/dL) ^b^	112 ± 18 ^a^	68 ± 11 ^b^	54 ± 9 ^b^	32 ± 6 ^c^	<0.001

^a^ One-way ANOVA with Tukey’s post hoc test; different superscripts within rows indicate *p* < 0.05. ^b^ Surrogate MAGE: surrogate mean amplitude of glycemic excursions, derived from sparse fasting-glucose measurements at seven time points (not the classical CGM-based MAGE; see Section 4.6). ^a–d^ Different superscript letters within the same row indicate statistically significant differences between groups at *p* < 0.05 by one-way ANOVA followed by Tukey’s post-hoc test; groups sharing ^a^ letter are not significantly different. Thus, ^c,d^ indicate statistically distinct post-hoc groupings where applicable. CV: coefficient of variation; SD: standard deviation; AUC: area under the curve; FG: fasting glucose.

**Table 4:** Selected Tukey’s Honest Significant Difference (HSD) post hoc comparisons (final body weight).

Comparison	Mean Diff	*p*-Value
Healthy Control vs. Diabetic Control	94 g	<0.0001
Diabetic Control vs. Diabetic + Onion	−38 g	0.004

**Table 5:** Oxidative stress markers in liver tissue at Day 60.

Parameter	Healthy Control	Diabetic Control	Onion Juice	Dapagliflozin	Combination
MDA (nmol/g tissue)	24.3 ± 3.1 ^a^	68.4 ± 7.2 ^b^	41.2 ± 4.8 ^c^	52.6 ± 5.3 ^d^	32.7 ± 3.6 ^e^
GSH (μmol/g tissue)	7.82 ± 0.64 ^a^	3.14 ± 0.42 ^b^	5.63 ± 0.51 ^c^	4.28 ± 0.39 ^d^	6.74 ± 0.58 ^e^
SOD (U/mg protein)	142 ± 11 ^a^	76 ± 8 ^b^	118 ± 10 ^c^	94 ± 9 ^d^	131 ± 12 ^e^
CAT (U/mg protein)	89 ± 7 ^a^	42 ± 5 ^b^	71 ± 6 ^c^	56 ± 5 ^d^	82 ± 7 ^e^

Different superscripts within rows indicate *p* < 0.05 by one-way ANOVA with Tukey’s post hoc test. MDA: malondialdehyde; GSH: reduced glutathione; SOD: superoxide dismutase; CAT: catalase.

**Table 6:** Adverse events and safety parameters during the 60-day treatment period.

Event	Healthy Groups (n = 39)	Diabetic Control (n = 10)	Diabetic + Onion (n = 10)	Diabetic + Dapa (n = 9)	Diabetic + Combo (n = 10)
Mortality	1 (2.6%) ^a^	0	0	1 (11.1%) ^a^	0
Mild hypoglycemia ^b^	0	0	1 (10%)	1 (11.1%)	2 (20%)
Polyuria	0	10 (100%)	8 (80%)	9 (100%)	10 (100%)
Soft stools	0	0	2 (20%)	0	2 (20%)
Weight loss > 10%	0	8 (80%)	3 (30%)	2 (22.2%)	1 (10%)
Genital infections	0	0	0	1 (11.1%)	1 (10%)

^a^ Deaths due to gavage-related complications. ^b^ Blood glucose 50–70 mg/dL. Dapa: dapagliflozin; Combo: combination therapy.

## Data Availability

All data supporting the findings of this study are available within the article and its Supplementary Materials.

## Supplementary Materials

The following supporting information can be downloaded at: https://www.mdpi.com/article/10.3390/ph19070999/s1, Table S1, Day 60 HbA1c summary statistics; Table S2, key pairwise comparisons (Tukey’s HSD, Day 60); Table S3, complete *p*-value matrix (Tukey’s HSD, Day 60); Table S4, change from baseline (Day 60–Day 0).

## Abbreviations

ACC	acetyl-CoA carboxylase
AMPK	AMP-activated protein kinase
AREs	antioxidant response elements
AUC	area under the curve
CaMKKβ	calcium/calmodulin-dependent protein kinase kinase β
CAT	catalase
CRTC2	CREB-regulated transcription coactivator 2
CV	coefficient of variation
DPP-4	dipeptidyl peptidase-4
DPPH	2,2-diphenyl-1-picrylhydrazyl
DTNB	5,5′-dithiobis-(2-nitrobenzoic acid)
EDTA	ethylenediaminetetraacetic acid
ELISA	enzyme-linked immunosorbent assay
ESI	electrospray ionization
FFAR	free fatty acid receptors
FOXO1	forkhead box O1
GAE	gallic acid equivalents
γ-GCL	γ-glutamylcysteine ligase
GLP-1	glucagon-like peptide-1
GLUT4	glucose transporter 4
GST	glutathione S-transferase
GSH	reduced glutathione
HbA1c	glycated hemoglobin
HO-1	heme oxygenase-1
HPLC-DAD	high-performance liquid chromatography–diode array detection
IC_50_	half-maximal inhibitory concentration
KATP	ATP-sensitive potassium channel
LKB1	liver kinase B1
MAGE	mean amplitude of glycemic excursions
MDA	malondialdehyde
MRM	multiple reaction monitoring
NF-κB	nuclear factor kappa B
NQO1	NAD(P)H quinone oxidoreductase 1
Nrf2	nuclear factor erythroid 2-related factor 2
ORAC	oxygen radical absorbance capacity
PEPCK	phosphoenolpyruvate carboxykinase
PYY	peptide YY
ROS	reactive oxygen species
SD	standard deviation
SGLT2	sodium-glucose cotransporter-2
SMCSO	S-methylcysteine sulfoxide
SOD	superoxide dismutase
SPCSO	S-propylcysteine sulfoxide
STZ	streptozotocin
T2DM	type 2 diabetes mellitus
TE	Trolox equivalents
UPLC-MS/MS	ultra-performance liquid chromatography–tandem mass spectrometry

## References

[1] Sun H., Saeedi P., Karuranga S., Pinkepank M., Ogurtsova K., Duncan B.B., Stein C., Basit A., Chan J.C.N., Mbanya J.C. (2022). IDF Diabetes Atlas: Global, Regional and Country-Level Diabetes Prevalence Estimates for 2021 and Projections for 2045. Diabetes Res. Clin. Pract..

[2] Lautsch D., Boggs R., Wang T., Gonzalez C., Milligan G., Rajpathak S., Malkani S., McLeod E., Carroll J., Higgins V. (2022). Individualized HbA1c Goals, and Patient Awareness and Attainment of Goals in Type 2 Diabetes Mellitus: A Real-World Multinational Survey. Adv. Ther..

[3] Huang L., Pan Y., Zhou K., Liu H., Zhong S. (2023). Correlation Between Glycemic Variability and Diabetic Complications: A Narrative Review. Int. J. Gen. Med..

[4] Heller S., Lingvay I., Marso S.P., Philis-Tsimikas A., Pieber T.R., Poulter N.R., Pratley R.E., Hachmann-Nielsen E., Kvist K., Lange M. (2020). Risk of Severe Hypoglycaemia and Its Impact in Type 2 Diabetes in DEVOTE. Diabetes Obes. Metab..

[5] Jarab A.S., Al-Qerem W.A., Hamam H., Heshmeh S.A., Al-Azzam S., Mukattash T.L., Alefishat E.A. (2023). Glycemic Control and Its Associated Factors among Diabetic Heart Failure Outpatients at Two Major Hospitals in Jordan. PLoS ONE.

[6] Sobanke A.O., Aiyeola A., Okwuonu F.I., Nnaemeka W.S., Ndubuisi J.C., Udeoji F.I., Adiele J.N. (2025). *Allium cepa* L. as a Natural Antioxidant: Its Efficacy in Combating Heat Stress-Induced Physiological Alterations. Hum. Nutr. Metab..

[7] Imran M., Kang H., Kim E.H., Lee S.G., Park H.M., Choi H., Kim S.H., Lee S., Oh S. (2025). Environmental and Genetic Effects on Phytochemical and Nutritional Composition of Onion (*Allium cepa* L.) Varieties in South Korea. Front. Plant Sci..

[8] Bedir A.S., Almasri R.S., Azar Y.O., Elnady R.E., Al Raish S.M. (2025). Exploring the Therapeutic Potential of *Allium cepa* and *Allium sativum* Extracts: Current Strategies, Emerging Applications, and Sustainability Utilization. Biology.

[9] Khalaf R.A. (2016). Exploring Natural Products as a Source for Antidiabetic Lead Compounds and Possible Lead Optimization. Curr. Top. Med. Chem..

[10] Alqudah S.M., Hailat M., Zakaraya Z., Abu Dayah A.A., Abu Assab M., Alarman S.M., Awad R.M., Hamad M.F., Vicaș L.G., Abu Dayyih W. (2024). Impact of Opuntia ficus-indica Juice and Empagliflozin on Glycemic Control in Rats. Curr. Issues Mol. Biol..

[11] Gupta A.J., Kaldate S., Volaguthala S., Mahajan V. (2025). Onion Nutritional and Nutraceutical Composition and Therapeutic Potential of Its Phytochemicals Assessed through Preclinical and Clinical Studies. J. Funct. Foods.

[12] Moon D.O. (2024). Plant-Derived Flavonoids as AMPK Activators: Unveiling Their Potential in Type 2 Diabetes Management through Mechanistic Insights, Docking Studies, and Pharmacokinetics. Appl. Sci..

[13] Lim S.H., Yu J.S., Lee H.S., Choi C.I., Kim K.H. (2021). Antidiabetic Flavonoids from Fruits of Morus alba Promoting Insulin-Stimulated Glucose Uptake via Akt and AMP-Activated Protein Kinase Activation in 3T3-L1 Adipocytes. Pharmaceutics.

[14] Singh A.K., Patel P.K., Choudhary K., Joshi J., Yadav D., Jin J.O. (2020). Quercetin and Coumarin Inhibit Dipeptidyl Peptidase-IV and Exhibit Antioxidant Properties: In Silico, In Vitro, Ex Vivo. Biomolecules.

[15] Liu H., Wang Y., Tong J., Li J., Ding H. (2024). Quercetin Analogs as α-Glucosidase Inhibitors with Antidiabetic Activity. Food Biosci..

[16] Günal-Köroğlu D., Catalkaya G., Yusufoğlu B., Kezer G., Esatbeyoglu T., Abd El-Aty A.M., Capanoglu E. (2025). Quercetin: Potential Antidiabetic Effects through Enzyme Inhibition and Starch Digestibility. Food Saf. Health.

[17] Dong B., Shi Z., Dong Y., Chen J., Wu Z.X., Wu W., Chen Z.S., Han C. (2022). Quercetin Ameliorates Oxidative Stress-Induced Cell Apoptosis of Seminal Vesicles via Activating Nrf2 in Type 1 Diabetic Rats. Biomed. Pharmacother..

[18] Hill C.R., Liu A.H., McCahon L., Zhong L., Shafaei A., Balmer L., Lewis J.R., Hodgson J.M., Blekkenhorst L.C. (2025). S-Methyl Cysteine Sulfoxide and Its Potential Role in Human Health: A Scoping Review. Crit. Rev. Food Sci. Nutr..

[19] Ali M., Hassan M., Ansari S.A., Alkahtani H.M., Al-Rasheed L.S., Ansari S.A. (2024). Quercetin and Kaempferol as Multi-Targeting Antidiabetic Agents against Mouse Model of Chemically Induced Type 2 Diabetes. Pharmaceuticals.

[20] Hu S., Lin C., Cai X., Zhu X., Lv F., Nie L., Ji L. (2022). The Urinary Glucose Excretion by Sodium–Glucose Cotransporter 2 Inhibitor in Patients with Different Levels of Renal Function: A Systematic Review and Meta-Analysis. Front. Endocrinol..

[21] Mosenzon O., Wiviott S.D., Cahn A., Rozenberg A., Yanuv I., Goodrich E.L., Murphy S.A., Heerspink H.J.L., Zelniker T.A., Dwyer J.P. (2019). Effects of Dapagliflozin on Development and Progression of Kidney Disease in Patients with Type 2 Diabetes: An Analysis from the DECLARE–TIMI 58 Randomised Trial. Lancet Diabetes Endocrinol..

[22] Wu J.Y., Hsu W.H., Kuo C.C., Tsai Y.W., Liu T.H., Huang P.Y., Chuang M.H., Hung K.C., Yu T., Lai C.C. (2025). A Retrospective Analysis of Combination Therapy with GLP-1 Receptor Agonists and SGLT2 Inhibitors versus SGLT2 Inhibitor Monotherapy in Patients with MASLD. Nat. Commun..

[23] Amawi H., Makhlouf T., Hammad A.M., Alsheyab S., Alhazaimeh R., Hall F.S., Khan J.T., Al-Trad B., Tiwari A.K. (2025). Sodium-Glucose Cotransporter-2 Inhibitor, Dapagliflozin, Reverses Depressive-like Behavior in a Mouse Model of Post-Traumatic Stress Disorder. Brain Res. Bull..

[24] Pei J., Wang X., Pei Z., Hu X. (2023). Glycemic Control, HbA1c Variability, and Major Cardiovascular Adverse Outcomes in Type 2 Diabetes Patients with Elevated Cardiovascular Risk: Insights from the ACCORD Study. Cardiovasc. Diabetol..

[25] Gabela A.M., Mthembu N., Hadebe S. (2026). Tryptophan Metabolism in Health and Disease—Implications for Non-Communicable Diseases. Immunol. Lett..

[26] Dhanya R., Arya A.D., Nisha P., Jayamurthy P. (2017). Quercetin, a Lead Compound against Type 2 Diabetes Ameliorates Glucose Uptake via AMPK Pathway in Skeletal Muscle Cell Line. Front. Pharmacol..

[27] Li Y.Q., Zhou F.C., Gao F., Bian J.S., Shan F. (2009). Comparative Evaluation of Quercetin, Isoquercetin and Rutin as Inhibitors of α-Glucosidase. J. Agric. Food Chem..

[28] Kim E.K., Kwon K.B., Song M.Y., Han M.J., Lee J.H., Lee Y.R., Lee J.H., Ryu D.G., Park B.H., Park J.W. (2007). Flavonoids Protect against Cytokine-Induced Pancreatic β-Cell Damage through Suppression of Nuclear Factor κB Activation. Pancreas.

[29] Sudarshan K., Aidhen I.S. (2017). Convenient Synthesis of 3-Glycosylated Isocoumarins. Eur. J. Org. Chem..

[30] Elosta A., Ghous T., Ahmed N. (2012). Natural Products as Anti-Glycation Agents: Possible Therapeutic Potential for Diabetic Complications. Curr. Diabetes Rev..

[31] Müller C., Gardemann A., Keilhoff G., Peter D., Wiswedel I., Schild L. (2012). Prevention of Free Fatty Acid-Induced Lipid Accumulation, Oxidative Stress, and Cell Death in Primary Hepatocyte Cultures by a Gynostemma pentaphyllum Extract. Phytomedicine.

[32] Ashcroft F.M. (2023). KATP Channels and the Metabolic Regulation of Insulin Secretion in Health and Disease: The 2022 Banting Medal for Scientific Achievement Award Lecture. Diabetes.

[33] Mi W., Hu Z., Xu L., Bian X., Lian W., Yin S., Zhao S., Gao W., Guo C., Shi T. (2022). Quercetin Positively Affects Gene Expression Profiles and Metabolic Pathway of Antibiotic-Treated Mouse Gut Microbiota. Front. Microbiol..

[34] Liu J., Liu Y., Huang C., He C., Yang T., Ren R., Xin Z., Wang X. (2025). Quercetin-Driven Akkermansia muciniphila Alleviates Obesity by Modulating Bile Acid Metabolism via an ILA/m6A/CYP8B1 Signaling. Adv. Sci..

[35] Chen K., Nakasone Y., Xie K., Sakao K., Hou D.X. (2020). Modulation of Allicin-Free Garlic on Gut Microbiome. Molecules.

[36] Guillamón E., Andreo-Martínez P., Mut-Salud N., Fonollá J., Baños A. (2021). Beneficial Effects of Organosulfur Compounds from *Allium cepa* on Gut Health: A Systematic Review. Foods.

[37] Tolhurst G., Heffron H., Lam Y.S., Parker H.E., Habib A.M., Diakogiannaki E., Cameron J., Grosse J., Reimann F., Gribble F.M. (2012). Short-Chain Fatty Acids Stimulate Glucagon-Like Peptide-1 Secretion via the G-Protein-Coupled Receptor FFAR2. Diabetes.

[38] Martin-Gallausiaux C., Marinelli L., Blottière H.M., Larraufie P., Lapaque N. (2021). SCFA: Mechanisms and Functional Importance in the Gut. Proc. Nutr. Soc..

[39] Zhang H.X., Li Y.Y., Liu Z.J., Wang J.F. (2022). Quercetin Effectively Improves LPS-Induced Intestinal Inflammation, Pyroptosis, and Disruption of the Barrier Function through the TLR4/NF-κB/NLRP3 Signaling Pathway In Vivo and In Vitro. Food Nutr. Res..

[40] El-Shaer N.O., Hegazy A.M., Muhammad M.H. (2023). Protective Effect of Quercetin on Pulmonary Dysfunction in Streptozotocin-Induced Diabetic Rats via Inhibition of NLRP3 Signaling Pathway. Environ. Sci. Pollut. Res..

[41] Wu J., Lv T., Liu Y., Liu Y., Han Y., Liu X., Peng X., Tang F., Cai J. (2024). The Role of Quercetin in NLRP3-Associated Inflammation. Inflammopharmacology.

[42] Ye Y., Bajaj M., Yang H.C., Perez-Polo J.R., Birnbaum Y. (2017). SGLT-2 Inhibition with Dapagliflozin Reduces the Activation of the NLRP3/ASC Inflammasome and Attenuates the Development of Diabetic Cardiomyopathy in Mice with Type 2 Diabetes. Further Augmentation of the Effects with Saxagliptin, a DPP4 Inhibitor. Cardiovasc. Drugs Ther..

[43] Chen H., Tran D., Yang H.C., Nylander S., Birnbaum Y., Ye Y. (2020). Dapagliflozin and Ticagrelor Have Additive Effects on the Attenuation of the Activation of the NLRP3 Inflammasome and the Progression of Diabetic Cardiomyopathy: An AMPK–mTOR Interplay. Cardiovasc. Drugs Ther..

[44] Kim S.R., Lee S.G., Kim S.H., Kim J.H., Choi E., Cho W., Rim J.H., Hwang I., Lee C.J., Lee M. (2020). SGLT2 Inhibition Modulates NLRP3 Inflammasome Activity via Ketones and Insulin in Diabetes with Cardiovascular Disease. Nat. Commun..

[45] Eid H.M., Nachar A., Thong F., Sweeney G., Haddad P.S. (2015). The Molecular Basis of the Antidiabetic Action of Quercetin in Cultured Skeletal Muscle Cells and Hepatocytes. Pharmacogn. Mag..

[46] Raz I., Jermendy G., Wilson P.W.F., Campaigne B.N., Strojek K., Kerr L., Kowalska I., Milicevic Z., Bozikov V., Jacober S.J. (2009). Effects of Prandial versus Fasting Glycemia on Cardiovascular Outcomes in Type 2 Diabetes: The HEART2D Trial. Diabetes Care.

[47] Sheng L., Yang G., Chai X., Zhou Y., Sun X., Xing Z. (2024). Glycemic Variability Evaluated by HbA1c Rather than Fasting Plasma Glucose Is Associated with Adverse Cardiovascular Events. Front. Endocrinol..

[48] Lian X.N., Zheng X.L., Zhu M.M., Li Z.H. (2025). Study on the Correlation between GDF-15 Levels and a Diagnostic Model for Diabetic Retinopathy. J. Diabetes Res..

[49] Papachristoforou E., Lambadiari V., Maratou E., Makrilakis K. (2020). Association of Glycemic Indices (Hyperglycemia, Glucose Variability, and Hypoglycemia) with Oxidative Stress and Diabetic Complications. J. Diabetes Res..

[50] Muscolo A., Maffia A., Marra F., Battaglia S., Oliva M., Mallamaci C., Russo M. (2025). Unlocking the Health Secrets of Onions: Investigating the Phytochemical Power and Beneficial Properties of Different Varieties and Their Parts. Molecules.

[51] Duan M., Feng J., Feng J.H., Wang X., Xiao X., He S., Guo H., Zhang W., Jiang Z., Wan T. (2025). Optimizing Processing Methods for Maximum Bioactive Retention: Comparative Metabolomic Analysis of Dried Loquat (*Eriobotrya japonica*) Flowers and Their Powdered Extracts. Front. Nutr..

[52] Guo H.H., Shen H.R., Wang L.L., Luo Z.G., Zhang J.L., Zhang H.J., Gao T.L., Han Y.X., Jiang J.D. (2023). Berberine Is a Potential Alternative for Metformin with Good Regulatory Effect on Lipids in Treating Metabolic Diseases. Biomed. Pharmacother..

[53] Ali M.A., Mahmoud S.A., Alkhedaide A., Soliman M.M., Al-Shafie T.A., El-Sayed Y.S., Shukry M., Ghamry H.I., Elblehi S.S. (2022). Boosting Effects of Cranberry and Cinnamaldehyde for Pioglitazone Amelioration of Liver Steatosis in Rat via Suppression of HIF-1α/Smad/β-Catenin Signaling. J. Funct. Foods.

[54] Mayyas A., Al-Samydai A., Oraibi A.I., Debbabi N., Hassan S.S., Al-Hussainy H.A., Salamatullah A.M., Dauelbait M., Bourhia M., Almaary K.S. (2025). Deciphering the Anti-Diabetic Potential of *Gymnema sylvestre* Using Integrated Computer-Aided Drug Design and Network Pharmacology. J. Cell. Mol. Med..

[55] Ghasemi A., Jeddi S. (2023). Streptozotocin as a Tool for Induction of Rat Models of Diabetes: A Practical Guide. EXCLI J..

[56] Raparelli V., Elharram M., Moura C.S., Abrahamowicz M., Bernatsky S., Behlouli H., Pilote L. (2020). Sex Differences in Cardiovascular Effectiveness of Newer Glucose-Lowering Drugs Added to Metformin in Type 2 Diabetes Mellitus. J. Am. Heart Assoc..

[57] Van Putten L.M. (1958). The Life Span of Red Cells in the Rat and the Mouse as Determined by Labeling with DFP32 In Vivo. Blood.

[58] Cohen R.M., Franco R.S., Khera P.K., Smith E.P., Lindsell C.J., Ciraolo P.J., Palascak M.B., Joiner C.H. (2008). Red Cell Life Span Heterogeneity in Hematologically Normal People Is Sufficient to Alter HbA1c. Blood.

[59] Higgins P.J., Garlick R.L., Bunn H.F. (1982). Glycosylated Hemoglobin in Human and Animal Red Cells: Role of Glucose Permeability. Diabetes.

[60] Pérez M., Dominguez-López I., Lamuela-Raventós R.M. (2023). The Chemistry Behind the Folin–Ciocalteu Method for the Estimation of (Poly)phenol Content in Food: Total Phenolic Intake in a Mediterranean Dietary Pattern. J. Agric. Food Chem..

[61] Ginet S.R., Gonzalez F., Marano M.L., Salecha M.D., Reiner J.E., Caputo G.A. (2025). Evaluation of the Ellman’s Reagent Protocol for Free Sulfhydryls under Protein Denaturing Conditions. Analytica.

[62] Percie du Sert N., Hurst V., Ahluwalia A., Alam S., Avey M.T., Baker M., Browne W.J., Clark A., Cuthill I.C., Dirnagl U. (2020). The ARRIVE Guidelines 2.0: Updated Guidelines for Reporting Animal Research. BMJ Open Sci..

[63] Furman B.L. (2021). Streptozotocin-Induced Diabetic Models in Mice and Rats. Curr. Protoc..

[64] Nair A., Jacob S. (2016). A Simple Practice Guide for Dose Conversion between Animals and Human. J. Basic Clin. Pharm..

[65] Lakhera P., Chaudhary V., Kush P., Kumar P., Ughade Y., Agrawal L., Patel G., Deshmukh K. (2025). Recent Advances in Glycated Hemoglobin Test Methods: From Lab to Point of Care Testing Devices. Int. J. Biol. Macromol..

[66] Zeb A., Ullah F. (2016). A Simple Spectrophotometric Method for the Determination of Thiobarbituric Acid Reactive Substances in Fried Fast Foods. J. Anal. Methods Chem..

[67] Li X. (2012). Improved Pyrogallol Autoxidation Method: A Reliable and Cheap Superoxide-Scavenging Assay Suitable for All Antioxidants. J. Agric. Food Chem..

[68] Hadwan M.H., Alta’ee A.H., Mohammed R.M., Hadwan A.M., Al-Kawaz H.S., Al Talebi Z.A. (2024). An Efficient Protocol for Quantifying Catalase Activity in Biological Samples. Bull. Natl. Res. Cent..

[69] Agbangba C.E., Sacla Aide E., Honfo H., Glèlè Kakai R. (2024). On the Use of Post-Hoc Tests in Environmental and Biological Sciences: A Critical Review. Heliyon.

[70] Service F.J., Molnar G.D., Rosevear J.W., Ackerman E., Gatewood L.C., Taylor W.F. (1970). Mean Amplitude of Glycemic Excursions, a Measure of Diabetic Instability. Diabetes.

[71] Baghurst P.A. (2011). Calculating the Mean Amplitude of Glycemic Excursion from Continuous Glucose Monitoring Data: An Automated Algorithm. Diabetes Technol. Ther..

