# Supplementary material for: Additive Effects of Quercetin-Rich Allium cepa L. Juice and Dapagliflozin on Glycemic Variability in Streptozotocin-Induced Diabetic Rats
Source: Pharmaceuticals (Basel). 2026 Jun 27;19(7):999. doi: 10.3390/ph19070999 (PMC13414573; doi:10.3390/ph19070999)
Supplement: Supplementary file 1 [file pharmaceuticals-19-00999-s001.zip › pharmaceuticals-4389762-supplementary.pdf]

# Supplementary Materials

*Additive Effects of Quercetin-Rich Allium cepa L. Extract and Dapagliflozin on Glycemic Variability in Streptozotocin-Induced Diabetic Rats*

## Tukey's HSD Statistical Analysis Results

### Statistical Methods

Post-hoc pairwise comparisons were performed using Tukey's Honest Significant Difference (HSD) test following one-way ANOVA. Tukey's HSD controls the family-wise error rate across all pairwise comparisons. All analyses were conducted using R (version 4.3.3). Statistical significance was set at $p < 0.05$.

**Table S1.** Day 60 HbA1c summary statistics.

| Grp | Treatment | Mean (%) | SD | SE | N |
|---|---|---|---|---|---|
| A | Healthy Control | 5.133 | 0.171 | 0.054 | 10 |
| B | Healthy + Onion | 4.893 | 0.158 | 0.050 | 10 |
| C | Healthy + Dapagliflozin | 4.952 | 0.215 | 0.068 | 10 |
| D | Healthy + Combination | 5.000 | 0.244 | 0.081 | 9 |
| E | Diabetic Control | 9.037 | 0.311 | 0.104 | 10 |
| F | Diabetic + Combination | 7.106 | 0.139 | 0.046 | 10 |
| G | Diabetic + Onion | 7.166 | 0.721 | 0.240 | 10 |
| H | Diabetic + Dapagliflozin | 7.115 | 0.459 | 0.145 | 9 |

SD: standard de*via*tion; SE: standard error; N: number of animals. Groups D and H each lost one animal to gavage-related complications.

**Table S2.** Key pairwise comparisons — Tukey's HSD (Day 60). Significance: *** $p < 0.001$; ns = not significant.

| Comparison | Mean Diff | p-value | Sig |
|---|---|---|---|
| Diabetic Control (E) vs. Healthy Control (A) | +3.904 | <0.001 | *** |
| Diabetic + Combo (F) vs. Diabetic Control (E) | −1.931 | <0.001 | *** |
| Diabetic + Onion (G) vs. Diabetic Control (E) | −1.871 | <0.001 | *** |
| Diabetic + Dapa (H) vs. Diabetic Control (E) | −1.922 | <0.001 | *** |
| Diabetic + Combo (F) vs. Diabetic + Onion (G) | +0.060 | 1.0000 | ns |
| Diabetic + Combo (F) vs. Diabetic + Dapa (H) | +0.009 | 1.0000 | ns |
| Diabetic + Onion (G) vs. Diabetic + Dapa (H) | −0.051 | 1.0000 | ns |

**Table S3.** Complete p-value matrix — Tukey's HSD (Day 60).

| | A | B | C | D | E | F | G | H |
|---|---|---|---|---|---|---|---|---|
| A | — | 0.788 | 0.942 | 0.991 | <0.001 | <0.001 | <0.001 | <0.001 |
| B | | — | 1.000 | 0.998 | <0.001 | <0.001 | <0.001 | <0.001 |
| C | | | — | 1.000 | <0.001 | <0.001 | <0.001 | <0.001 |
| D | | | | — | <0.001 | <0.001 | <0.001 | <0.001 |
| E | | | | | — | <0.001 | <0.001 | <0.001 |
| F | | | | | | — | 1.000 | 1.000 |
| G | | | | | | | — | 1.000 |
| H | | | | | | | | — |

Groups: A, Healthy Control; B, Healthy + Onion; C, Healthy + Dapagliflozin; D, Healthy + Combination; E, Diabetic Control; F, Diabetic + Combination; G, Diabetic + Onion; H, Diabetic + Dapagliflozin. The matrix is symmetric; only the upper triangle is shown.

**Table S4.** Change from baseline (Day 60 − Day 0) in HbA1c.

| Grp | Treatment | Mean Change (%) | SD |
|---|---|---|---|
| A | Healthy Control | −0.061 | 0.078 |
| B | Healthy + Onion | −0.096 | 0.343 |

| Grp | Treatment | Mean Change (%) | SD |
|---|---|---|---|
| C | Healthy + Dapagliflozin | −0.366 | 0.223 |
| D | Healthy + Combination | −0.353 | 0.140 |
| E | Diabetic Control | +3.998 | 0.375 |
| F | Diabetic + Combination | +2.086 | 0.224 |
| G | Diabetic + Onion | +2.097 | 0.828 |
| H | Diabetic + Dapagliflozin | +2.195 | 0.449 |

### Key Findings

1. All three treatment modalities (onion, dapagliflozin, combination) significantly reduced HbA1c compared with the diabetic control (all $p < 0.001$).
2. No significant differences were found among the three diabetic treatment groups (F vs. G: $p = 1.000$; F vs. H: $p = 1.000$; G vs. H: $p = 1.000$).
3. The combination therapy (F) showed numerically lower HbA1c (7.106%) than the monotherapies, but this difference was not statistically significant.
4. These findings support an additive rather than a synergistic effect, as the combination did not produce significantly greater HbA1c reduction than monotherapy.
5. While endpoint HbA1c values were comparable across treatment groups, the combination therapy demonstrated significantly lower glycemic variability (CV-HbA1c: 3.9 ± 0.6%) than the monotherapies, as detailed in the main manuscript (Table 3).
